# Expect the unexpected: fulminant myocardial cytotoxic Injury from Trabectedin

**DOI:** 10.1186/s40959-024-00257-7

**Published:** 2024-10-15

**Authors:** Annie J. Tsay, Mohan Satish, Elizabeth Corley, Ashley Ezema, Neisha DeJesus, Stephen Wisely, Eileen McAleer, Chen Zhang, Su Yuan, Edwin Homan, Jennifer E. Liu, Jonathan W. Weinsaft, Sandra D’Angelo, Stephanie A. Feldman, Angel T. Chan

**Affiliations:** 1 Memorial Sloan Kettering Cancer Center | Weill Cornell Medicine, 1275 York Ave, New York, NY 10065 USA; 2 New York-Presbyterian Weill Cornell Medicine, New York, NY USA; 3 Weill Cornell Medical College, New York, USA

**Keywords:** Trabectedin, Cardiotoxicity, Cardio-oncology, Chemotherapy, Cancer, Sarcoma

## Abstract

**Background:**

Trabectedin (Tbt) is an alkylating agent prescribed for soft tissue sarcomas after treatment failure of first line agents. While cardiomyopathy can occur with Tbt treatment after anthracycline exposure, Tbt-induced fulminant myocardial cytotoxic injury in the setting of other systemic cytotoxicity associated with Tbt has not been reported.

**Case presentation:**

51-year-old female with hypertension, hyperlipidemia, metastatic leiomyosarcoma with progression of disease despite several lines of chemotherapy including doxorubicin-based therapy was started on Trabectedin (Tbt) 5 days prior to presentation with symptoms of fever, myalgias, arthralgias, and palpitations. She was admitted for management of rhabdomyolysis, acute kidney and liver injuries which were reportedly known to be associated with Tbt treatment. A baseline electrocardiogram (ECG) revealed sinus tachycardia with non-specific T-wave changes, and a transthoracic echocardiogram (TTE) was unremarkable. However, on day 3 of hospitalization, an episode of asymptomatic sustained monomorphic ventricular tachycardia with a heart rate of 150 beats per minute was captured on telemetry. A 12-lead ECG revealed new septal T-wave inversions. Labs revealed rising hs-TnI levels (peak at 37,933ng/L) and serum markers suggested multi-organ failure. Steroids were initiated given its role in treating multi-organ Tbt-induced toxicity. A cardiac MRI to rule out myocarditis and left heart catheterization to rule out obstructive coronary artery disease were forgone due to acute renal failure. A right heart catheterization with an endomyocardial biopsy was performed revealing normal cardiac filling pressures and indices. Pathology showed cytoplasmic vacuoles indicating drug-induced myocardial cytotoxicity. Serial echocardiograms revealed preserved biventricular function. The patient’s clinical condition deteriorated with multi-organ failure despite maximal supportive care in the intensive care unit. She ultimately passed away, and an autopsy was declined.

**Conclusion:**

This is the first reported case of fulminant myocardial injury after initiation of Tbt with histologic evidence of drug-induced myocardial cytotoxicity. While it is unclear if anthracyclines potentiate Tbt cytotoxic injury as in this case, it is plausible; and that Tbt-induced cardiotoxicity ranges from subclinical to fulminant. Given increasing use of Tbt in refractory high-grade sarcomas, raising awareness of its toxicity profile will improve early detection and outcomes.

## Background

Trabectedin (Tbt) is an alkylating agent approved by the FDA in 2015 for high-grade soft tissue sarcomas (STS) after treatment failure of first line agents such as doxorubicin [1]. It binds to the minor groove of DNA affecting both transcription and repair mechanisms independent of the p53 apoptotic pathway [2]. Cardiomyopathy with or without associated heart failure can occur among those treated with anthracyclines followed by Tbt [3, 4]. A post-marketing analysis of ten phase II and III studies evaluating the cardiac safety of Tbt after doxorubicin therapy for STS reported an incidence of cardiomyopathy at 3.7% and HF at 2.9% with a median cumulative prior anthracycline dose of 329.75mg/m^2^ [5]. Among those with prior chemotherapy treatment including doxorubicin and ifosfamide, Tbt has been documented to have a remarkable median progression free survival of 4.3 months compared to 1.6 months among those treated with dacarbazine with a HR 0.55 (95% CI: 0.42–0.73) favoring Tbt compared to dacarbazine [6]. Grade 3 and 4 toxicities from Tbt have included transaminitis, elevated creatinine phosphokinase, and rhabdomyolysis [6]. Treatment-related death from Tbt include sepsis/septic shock, rhabdomyolysis, renal failure, cardiac arrest, and multiorgan failure [6]. While LV systolic dysfunction and HF symptoms have been well documented among those who have received high doses of doxorubicin (> 350mg/m^2^), multiorgan involvement is not a known side effect [7]. LV systolic dysfunction and HF among those treated with both anthracyclines and Tbt have been previously reported and hypothesized to be attributed to Tbt toxicity, but pathologic evidence has not been used to support those findings. The case that we present raises concern that Tbt toxicity may be synergistic with prior anthracycline exposure and/or a separate entity based on pathologic evidence suggestive of fulminant myocardial injury from drug-induced cytotoxic effects of Tbt.

## Case presentation

**Fig. 1:**
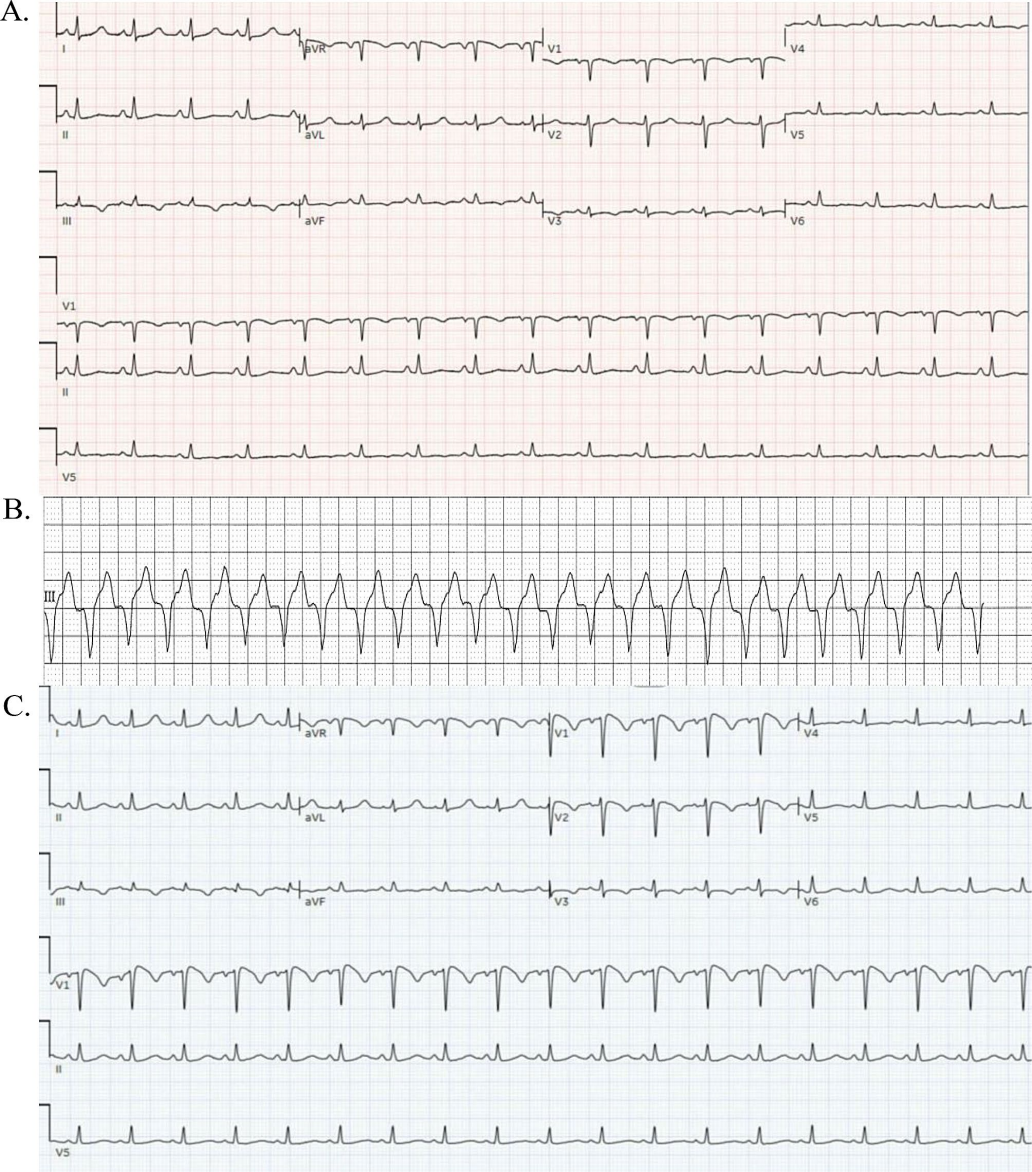
Electrocardiograms (ECGs) and telemetry tracing. **(A)** A 12-lead ECG on admission with sinus tachycardia and non-specific ST-T wave abnormalities, **(B)** Telemetry with sustained wide complex tachycardia), **(C)** ECG with new septal T wave inversions in leads V1-V3

We present a 51-year-old female with hypertension, hyperlipidemia, metastatic leiomyosarcoma with progression of disease despite several lines of chemotherapy including gemcitabine, docetaxel, and doxorubicin (total cumulative dose: 444 mg/m^2^; liposomal form 79 mg/m^2^) started 8 months prior and discontinued 1 month prior to initiation of third-line agent, Trabectedin (Tbt). Baseline serum laboratory tests were normal with serum blood urea nitrogen (BUN) 9 mg/dL, creatinine 0.8 mg/dL, aspartate aminotransferase (AST) 23 U/L, alanine aminotransferase (ALT) 26 U/L, and creatinine kinase (CK) 30 U/L. Five days after initiation of Tbt, the patient presented to the urgent care center with symptoms including fever, myalgias, arthralgias, and palpitations. On admission, her vital signs included a temperature of 99.3^◦^F, heart rate of 113 BPM, blood pressure of 106/72 mmHg, respiratory rate of 20, and oxygen saturation of 95% on room air. Her exam was notable for tachycardia and right upper quadrant pain. She was found to have an acute kidney injury (AKI) with a serum creatinine of 2.7 mg/dL (baseline 0.8 mg/dL) and transaminitis with alanine transaminase (ALT) of 734 U/L and aspartate aminotransferase (AST) of 690 U/L. Creatinine kinase (CK) was normal at 64 U/L (normal 26–140 U/L). Cardiac high sensitivity troponin-I (hs-TnI) was mildly elevated at 90.0 ng/L, while brain natriuretic peptide (BNP) was normal at 32 pg/mL. A 12-lead electrocardiogram (ECG) on admission revealed sinus tachycardia with non-specific ST-T wave changes (Fig. 1a). Transthoracic echocardiogram (TTE) revealed normal biventricular function with a left ventricular ejection fraction (LVEF) of 65% without regional wall motion abnormalities or valvulopathy. She was admitted for evaluation of a new AKI and transaminitis.

On day 3 of admission, she had a 52 s episode of asymptomatic sustained monomorphic ventricular tachycardia at 150 bpm noted on telemetry (Fig. 1b). She reported atypical right sided pleuritic chest discomfort. A repeat 12-lead ECG revealed new septal T wave inversions in leads V1-V3 (Fig. 1c), and hs-TnI increased from 330ng/L to 1,950 ng/L on day 5, which peaked at 37,933 ng/L on day 7 of admission. Repeat BNP rose to 123 pg/mL. A repeat TTE on day 6 showed preserved LV systolic function. Additional labs were notable for worsening CK 1,617 U/L and elevated C-reactive protein (CRP) to 9.52 mg/dL (normal ≤ 0.50 mg/dL). Prednisone 1 mg/kg was started on day 5 for rising liver enzymes concerning for Tbt-induced liver toxicity. Pulse-dosed steroids 1 g IV for 3 days was started on day 6 for presumed myocarditis vs. myocardial injury from Tbt in the setting of rising cardiac enzymes.

**Fig. 2:**
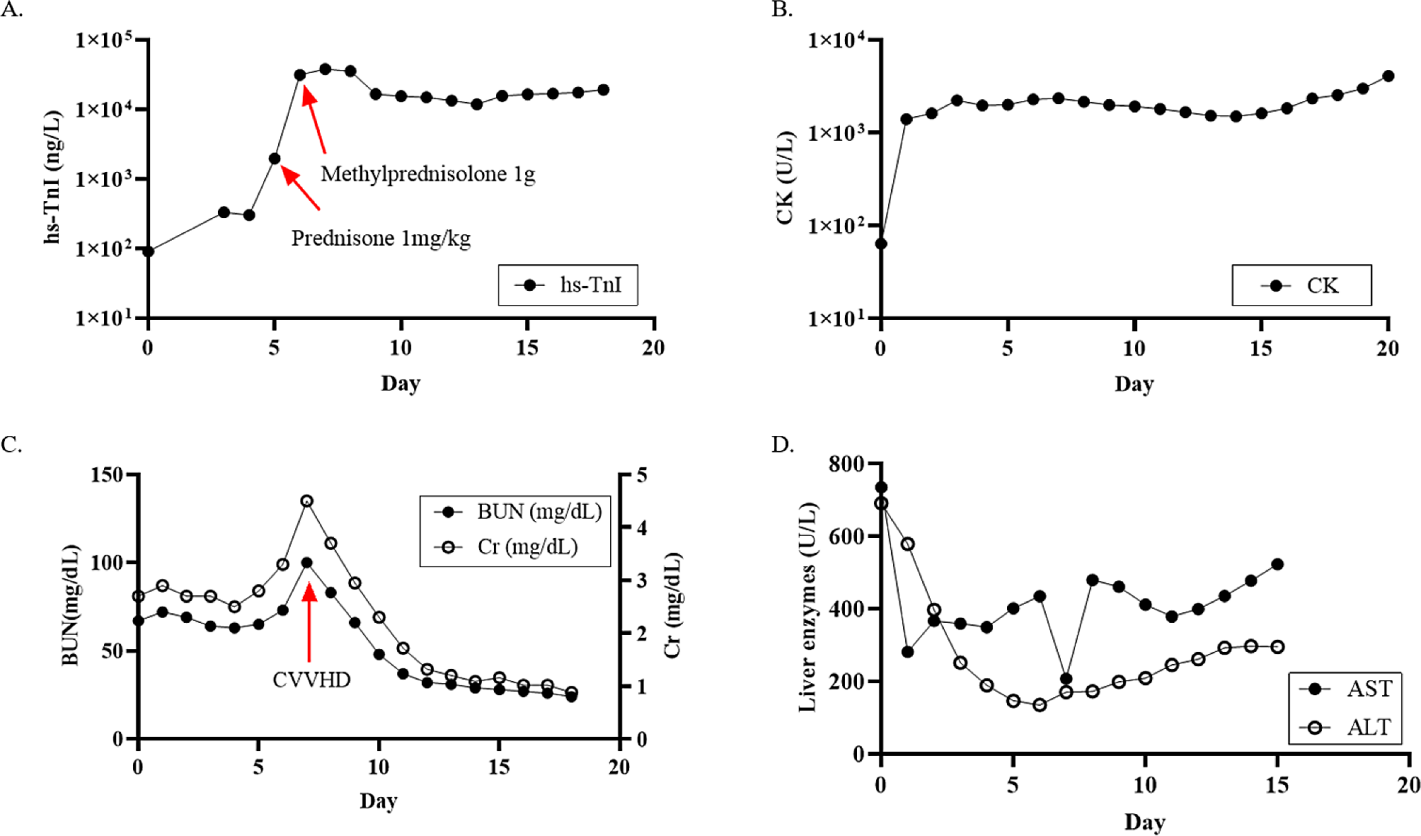
Laboratory trends for multi-organ involvement from Trabectedin-induced cytotoxicity. **(A)** Elevated hs-TnI (ng/L) after Tbt initiation and steroid treatment (arrows). **(B)** Rhabdomyolysis with elevated total CK. **(C)** Acute renal failure with elevated BUN/Cr. **(D)** Acute liver failure with elevated AST/ALT

**Fig. 3:**
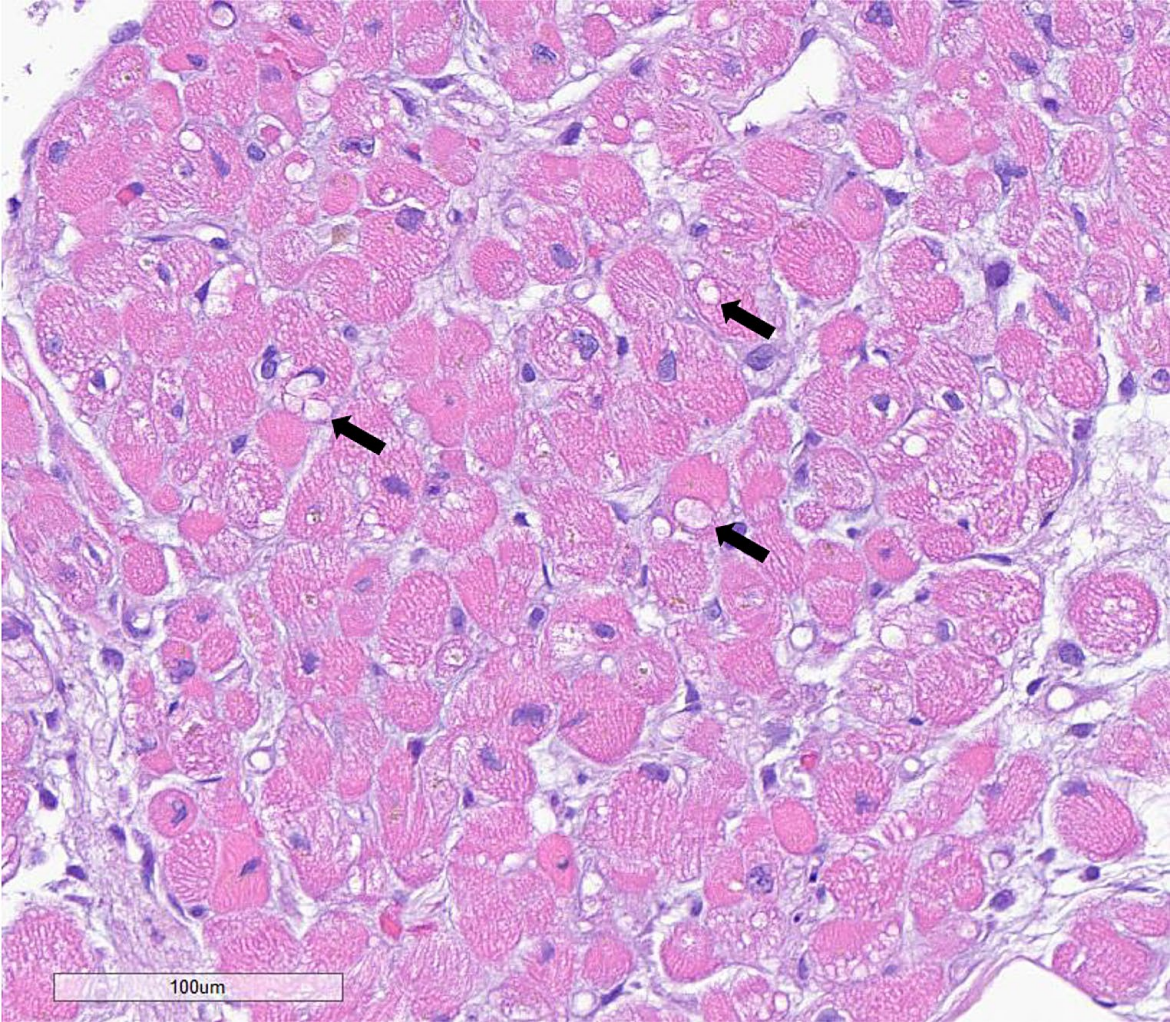
H&E stain of Endomyocardial biopsy (200x). After one dose of steroids with cytoplasmic vacuolations (arrows) suggestive of drug-induced myocardial injury

An infectious work-up including blood cultures, extended respiratory virus panel, and gastroenterology panel were unremarkable. An ischemic evaluation based on new T wave inversions was considered, but a left heart catheterization (LHC) to rule out acute coronary syndrome (ACS) and a cardiac MRI for myocarditis and scar were deferred due to AKI and low suspicion for ACS given unchanged TTE and serial ECGs without dynamic changes. A right heart catheterization (RHC) with endomyocardial biopsy was performed and revealed normal pressures and cardiac indices (right atrial pressure (RAP) of 5 mmHg, RV pressure of 32/3 mmHg, PA pressure 29/16 mmHg (mean 20 mmHg), pulmonary capillary wedge pressure (PCWP) 8 mmHg measured at end expiration, cardiac output (CO) 5.88 L/min by estimated Fick method, cardiac index (CI) 2.93 L/min/m^2^ by estimated Fick method, pulmonary vascular resistance (PVR) 2.04WU). An endomyocardial biopsy was obtained on day 6 of admission after a single dose of pulse-dosed steroids 1 g had been administered with no effect on hs-TnI levels, which continued to rise from 1,950 to 31,240 ng/L (Fig. 2a). However, on day 7, hs-TnI levels finally peaked at 37,933ng/L. During this time, she became anuric on day 7 requiring continuous veno-venous hemodialysis (CVVHD) with elevated CK and BUN/Cr (Fig. 2b, c). An abdominal ultrasound to evaluate causes for hyperbilirubinemia to 6.5 mg/dL and transaminitis (AST 522 U/L, ALT 295 U/L) revealed new liver masses, patent vasculature, and no biliary dilatation (Fig. 2d). The pathology report of the endomyocardial biopsy resulted on day 8 of admission revealing diffuse cytoplasmic vacuolations concerning for medication-induced cytotoxic effects without evidence of immune cell infiltration to suggest acute myocarditis (Fig. 3). Additional findings on the hematoxylin & eosin (H&E) stain revealed nuclear densities to be within normal limits without an increase in myocyte apoptosis or necrosis (Fig. 3). Pulse dosed steroids at 1 g IV methylprednisolone was continued for a total of 3 days before transitioning to oral weight-based (1 mg/kg) prednisone dosing on day 9 corresponding with an improvement of hs-TnI levels (range 11,768 − 19,133 ng/L) (Fig. 2a). Serial TTEs during the hospitalization remained unchanged with preserved biventricular function. However, her course was further complicated by sepsis, likely from an intraabdominal source, and multiorgan failure leading to a transition to comfort measures. The patient passed away and an autopsy was declined.

## Discussion

This is the first reported case of suspected biopsy-proven Tbt-induced myocardial injury with prior anthracycline exposure demonstrating severe multi-organ failure including rhabdomyolysis, renal, and liver failure. Tbt (Yondelis) is an alkylating agent used to treat soft tissue sarcomas after failure of first line agents (e.g., anthracyclines and ifosfamide) [6]. Laboratory and clinical trials report a synergistic effect between Tbt and anthracyclines, platinum-based agents, and DNA topoisomerase inhibitors [5, 8]. Tbt binds to the minor groove of the DNA double helix and interferes with DNA repair and transcription pathways halting the cell cycle at the G2 phase as a hallmark of its cytotoxic activity leading to cellular apoptosis [1, 9]. It modulates the production of cytokines and chemokines by macrophages in the tumor microenvironment [1]. Previously reported side effects include rhabdomyolysis and hepatotoxicity as observed in our case [10]. Steroids may be used for pre-medication to reduce liver and bone marrow toxicity in advanced sarcoma [11] as was performed in this case.

The cardiotoxic profile of Tbt in soft tissue sarcomas with prior anthracycline exposure has been reported in randomized clinical trials (RCTs) [6] and case series [4, 12] inferred from timing of Tbt exposure without pathologic evidence. RCTs revealed “no cumulative cardiotoxicity” with Tbt and the incidence rate of cardiac adverse events (CAEs) including cardiomyopathy, arrhythmias, palpitations was 0.2–3.3% [6, 12]. Case reports of individuals with prior anthracycline exposure subsequently on Tbt revealed profound cardiomyopathy LVEF ≤ 35% with NYHA Functional Class III symptoms, or myocardial infarction as early as 1-week after Tbt initiation [4, 12]. Prior studies extrapolated Tbt-related toxicity based purely on a temporal relationship and known side effect profile [4, 12]. Our case, provides histologic evidence to support that there may be a component of direct myocardial cytotoxic injury from Tbt with possible potentiation from prior high-dose anthracycline exposure though, in our case, without evidence of cardiomyopathy by serial TTEs. Despite markedly elevated troponin levels, lack of immune cell infiltration in the myocardium and presence of myocardial cytoplasmic vacuolation in our case suggest direct cytotoxic injury of the myocardium from serial exposure of anthracycline followed by Tbt rather than an immune-related adverse event [13]. Data supporting Tbt-induced cytotoxicity include close temporal relationship to Tbt initiation, acute multi-organ involvement (not characteristic of anthracycline exposure but known to occur with Tbt), and pathologic evidence of cytoplasmic vacuolation (known feature of drug-induced cytotoxicity) that may result in apoptosis or cell death with possible potentiation from prior high-dose anthracycline exposure [14]. While the mechanistic difference between Tbt-induced cardiomyopathy and direct cytotoxic injury to the myocardium has not been reported it is plausible that it falls on a spectrum from subclinical to deadly. We hypothesize that the mechanism behind the synergistic effect of prior doxorubicin exposure followed by Tbt treatment may be due to increased cellular permeability leading to influx of Tbt and resultant accumulation of intracellular Tbt leading to increased cytotoxicity, cellular apoptosis, and multiorgan failure. This hypothesis is supported by an in vitro study using two human soft tissue sarcoma cell lines to investigate the cytotoxic effects of Tbt, doxorubicin, and platinum-based therapies when used as monotherapy, concurrently, or sequentially [8]. The study found that combination doxorubicin and Tbt resulted in approximately 5-fold increase in cellular apoptosis compared to doxorubicin monotherapy (11% versus 2%) [8]. Additionally, when cells were pre-treated with Tbt for 24 h, there was a significant increase in doxorubicin levels intracellularly by approximately 30% compared to cells without pre-treatment (*p* = 0.006) [8]. This suggests that combination or sequential therapy of doxorubicin and Tbt may cause a priming effect with increased cellular permeability resulting in higher levels of drug accumulation intracellularly making the environment more susceptible to cytotoxicity and cellular apoptosis once the concentration surpasses a threshold. While this study focused on the sequence of treatment with Tbt first followed by doxorubicin, the overall study revealed that the use of Tbt and doxorubicin may result in synergistic enhancement of cytotoxicity irrespective of treatment sequence.

Based on these findings, Tbt-induced myocardial cytotoxic injury can be life-threatening particularly among those with prior anthracycline exposure requiring early recognition through collaborative efforts between cardiologists and oncologists, discontinuation of Tbt, supportive care, and early initiation of steroids in severe cases.

## Conclusions

Direct cytotoxic injury to myocardium without evidence of cardiomyopathy from Tbt is rare. To our knowledge, this is the first case of biopsy proven drug-induced myocardial injury after recent Tbt treatment. Given increasing use of Tbt as a second- or third-line agent for the treatment of high-grade sarcomas, a high clinical suspicion for Tbt-induced cardiotoxicity particularly among those with prior anthracycline exposure will likely be encountered more frequently, therefore raising awareness to ensure early recognition is of critical importance.

## Data Availability

No datasets were generated or analysed during the current study.

## Abbreviations

ACS: Acute coronary syndrome
ALT: Alanine transaminase
AST: Aspartate aminotransferase
BNP: Brain natriuretic peptide
CI: Cardiac index
CO: Cardiac output
CVVHD: Continuous veno-venous hemodialysis
DNA: Deoxyribonucleic acid
ECG: Electrocardiogram
hs-TnI: High sensitivity cardiac troponin-I
LHC: Left heart catheterization
LV/LVEF: Left ventricular ejection fraction
PCWP: Pulmonary capillary wedge pressure
PVR: Pulmonary vascular resistance
RA: Right atrium
RHC: Right heart catheterization
RV: Right ventricle
Tbt: Trabectedin
TTE: Transthoracic echocardiogram
WU: Woods Units
